# Magnitude of Malaria-Typhoid Fever Coinfection in Febrile Patients at Arba Minch General Hospital in Southern Ethiopia

**DOI:** 10.1155/2022/2165980

**Published:** 2022-08-01

**Authors:** Sifray Batire, Tsegaye Yohanes, Dagimawie Tadesse, Melat Woldemariam, Befikadu Tariku, Zebenay Sanbeto, Debalke Dale, Dagninet Alelign

**Affiliations:** ^1^Department of Medical Laboratory Science, College of Medicine and Health Sciences, Arba Minch University, Arba Minch, Ethiopia; ^2^School of Public Health, College of Medicine and Health Sciences, Arba Minch University, Arba Minch, Ethiopia; ^3^Department of Pharmacy, College of Medicine and Health Sciences, Arba Minch University, Arba Minch, Ethiopia

## Abstract

**Background:**

Coinfection with malaria and typhoid fever is a major public health issue in developing countries. In endemic areas, including Ethiopia, people are at risk of acquiring both malaria and typhoid fever at the same time. Therefore, this study aimed to determine the magnitude of malaria-typhoid fever coinfection in febrile patients attending hospital at Southern Ethiopia.

**Methods:**

A hospital-based cross-sectional study was carried out on 416 febrile patients attending Arba Minch General Hospital from 1^st^ October to 30^th^ December 2021. The data was collected using a pretested structured questionnaire. Capillary and Venus blood samples were collected for assessing malaria and typhoid fever, respectively. Blood smear, culture, and biochemical tests were performed based on standard parasitological and microbiological methods. The *P-*value ≤ 0.05 was considered statistically significant.

**Results:**

The magnitude of malaria, typhoid fever, and their coinfections was 26.2% (109/416), 6.5% (27/416), and 3.1% (13/416), respectively. Among the confirmed malaria cases, about 66% of infections were *Plasmodium falciparum*. The malaria-typhoid fever coinfection showed a statistically significant association with a clinical presentation of a continuous pattern of fever (AOR = 5.84; 95% CI: 1.44–23.71, *P* = 0.014) and chills (AOR = 3.94; 95% CI: 1.04–14.89, *P* = 0.044). About 29.6% of *Salmonella* isolates were multidrug-resistant (MDR).

**Conclusion:**

The total rate of coinfection with malaria and typhoid fever was comparable to that of previous studies. With the consideration of higher prevalence of drug resistance of *Salmonella* spp. and higher prevalence of malaria‐typhoid fever coinfection, proper diagnostic procedure should be implemented for proper use of drugs.

## 1. Background

Malaria and typhoid fever are the most frequent acute febrile infections with no discernible symptoms and leading causes of morbidity and mortality in sub-Saharan Africa [1–5]. Acute febrile illness is defined as a rise in body temperature above the normal range of 36.5–37.5°C [2, 6–8]. Malaria and typhoid fever are caused by quite distinct organisms, protozoa, and Gram-negative bacilli, respectively, and are spread by separate routes [9,10].

Malaria is the world most complex and overwhelming health problem, affecting the vast majority of tropical and subtropical regions [2,11]. *Plasmodium species* are known single-celled protozoan parasites that cause malaria in humans [12,13]. In 2019, there were 229 million cases of malaria globally and 405,000 deaths despite massive efforts for malaria control. More than 90% of all malaria deaths worldwide are attributed to Sub-Saharan African countries [11,12,14,15]. In Ethiopia, approximately 68% of the land mass of the country have favorable conditions for malaria transmission, and 60% of the population are at risk [11]. *P. falciparum* is the most virulent and is responsible for 1.4 to 2.6 million deaths worldwide [10].

Typhoid fever is a bacterial infection caused by *Salmonella* bacteria, which cause around 20 million cases per year globally, and severe cases may lead to serious complications or even death [4–9,16]. *Salmonella* is a Gram-negative bacillus found in nature predominantly as parasites of man and other animals' intestinal tracts. Serologic markers on polysaccharide somatic (O) and protein flagella (H) antigens distinguish *Salmonella* subspecies [17–19]. *Salmonella typhi* and the *paratyphoid* bacilli are only found in the intestines of humans, where they have a high level of pathogenicity and frequently cause invasive disease [16,20]. It is transmitted mainly by the fecal–oral route through contaminated water and food [11–14]. The most common clinical presentation of typhoid fever includes different grade of fever, headache, fatigue, malaise, loss of appetite, cough, constipation, and skin rash [16,17,21,22]. Despite its clinical importance, laboratory diagnosis of typhoid fever in resource-limited countries widely depends on the Widal test, which has low specificity and positive predictive value [17,22]. Hence, it leads to wrong diagnoses and overantibiotic prescriptions that further increase the emergence of multidrug-resistant strains of commonly used drugs [17,19,22].

Malaria and typhoid coinfection can cause major complications and illnesses, mainly maternal and childhood anemia, fever, miscarriage, stillbirth, and even death. In terms of signs and symptoms, malaria and typhoid coinfections have a great deal in common. And usually, febrile patients are frequently misdiagnosed due to overlap of the same signs and symptoms, which are the main barriers to treating these diseases, besides true coinfections with malaria and typhoid fever [4,7,14].

Malaria may increase the risk of bacterial superinfection by affecting the immune system. Under severe malaria conditions, such as hemolysis and impaired leukocyte and macrophage activity due to parasite phagocytosis and subsequent malaria pigment build-up and immunosuppression, the invading bacteria grow and cause bacteremia [6–8].

Despite the importance of concurrent malaria and typhoid fever in the tropics, the diagnostic problems and public health implications as well as the prevalence of coinfection and its related factors and clinical conditions have not been thoroughly studied in most African countries, including Ethiopia, where the prevalence of both infections is expected to be high [3,4,11]. Considering that malaria and typhoid fever are endemic in Ethiopia and the prevalence of these diseases varies with demographic, environmental, and climatic conditions, updated information regarding the epidemiology of coinfection of malaria-typhoid fever in the study area may aid in designing appropriate treatment and intervention strategies [14,17,19,22]. Therefore, this study aimed to determine the magnitude of malaria-typhoid fever coinfection in febrile patients attending Arba Minch General Hospital, Southern Ethiopia.

## 2. Materials and Methods

### 2.1. Study Design and Area

A hospital-based cross-sectional study was conducted at Arba Minch General Hospital, southern Ethiopia, from 1^st^ October to 30^th^ December 2021. Arba Minch is located in southern Ethiopia, 505 kilometers from Addis Ababa, the country's capital. The altitude of the town is 1,285 m above sea level. The study area is mostly endemic to malaria, and diagnosis and treatment of febrile patients are among the hospital's routine services.

### 2.2. Sample Size and Sampling Technique

The study participants consist of randomly selected febrile patients who came to the outpatient clinic of Arba Minch General Hospital with clinically suspected malaria and/or typhoid fever. The sample size was calculated using a single population proportion formula with 5% margin of error, 95% confidence level, and 48.3% prevalence *Plasmodium falciparum* from the previous study [14]. With the consideration of 10% nonresponse rate, the total sample size was 416. The systematic random selection method was followed by calculating the K^th^ value (interval). The first subject was recruited by lottery method, and then, every K^th^ febrile patient was included in the study. Febrile patients aged greater than 2 years old were included, while patients who had taken antimalarial drugs and/or antibiotics within the previous two weeks were excluded.

### 2.3. Data Collection

The data were collected by three trained diploma nurses and three medical laboratory professionals. Sociodemographic characteristics (age, sex, residence, level of education, marital status, and occupation) and common clinical and behavioral factors that can be potentially a risk and/or indicator for malaria-typhoid coinfection were collected by a pretested structured questionnaire administered through a face-to-face interview. Capillary and venous blood samples were collected by a trained medical laboratory professional at Arba Minch General Hospital from patients. Capillary blood specimens were collected from finger pricks aseptically using a sterile blood lancet. A total of 10 ml and 4 ml of blood samples were collected aseptically using 70% alcohol from a peripheral vein of adult and pediatric febrile patients, respectively.

### 2.4. Sample Processing

Capillary blood specimens were used to prepare thick and thin blood film smears for isolation and identification of malaria. In brief, the smears were air dried. Thin films were fixed with methanol, and both thin and thick films were stained with 10% Giemsa stain for 10 minutes [11]. All dried slides were placed in slide boxes and examined for malaria. The presence of malaria parasites on thick blood smear and the identification of *Plasmodium* species from smear was done through an oil-immersed objective (100×), at 1000× magnification. The thick smear was used to determine whether the malaria parasites were present or absent, and the thin smear was used to identify the type of *Plasmodium* species. During the microscopic examination, a slide was regarded as negative after 200 fields had been examined without finding a *Plasmodium* parasite by two laboratory technologists. To assure the quality of the microscopic examinations, all positive and 10% of the negative slides were reexamined blindly by a third reader to remove discrepant results.

Venous blood samples were used for the isolation and identification of *Salmonella* spp. Half a milliliter of collected blood was dispensed into a sterile bottle containing 45 milliliters of Tryptic soy broth culture medium (HiMedia, India) and 18 milliliters of Tryptic soy broth culture medium (HiMedia, India) in a 1 : 10 ratio. The inoculated bottles were incubated aerobically at 37°C for 7 days in the Microbiology and Parasitology Laboratory at Arba Minch University's College of Medicine and Health Sciences and observed for signs of bacterial growth (turbidity, hemolysis, air bubbles or gas production, and clot formation) on daily basis for up to 7 days. Bottles that showed signs of growth were further processed by Gram stained and subcultured on MacConkey agar (Park Scientific Unlimited-England) at 37°C for 24 hrs. The plate was then aerobically incubated for 18–24 hrs at 37°C. A blood sample containing broth with no bacterial growth after 7 days was subcultured on blood agar before being reported as a negative result. Identification of isolates was done by colony morphology, Gram staining, and biochemical tests using Triple Sugar Iron Agar (TSI) (Becton, Dickinson, USA), Motility, Lysine Iron Agar (LIA) (Liofilchem-Italy), Citrate Recovery Identified Screening Test (OXID LTD, England), Urease Test (Mast Group Ltd, UK), Indole test, and for species identification, agglutination with *Salmonella*-specific antisera (Welcome Diagnostics, Dartford, UK) was used [22–24].

### 2.5. Antimicrobial Susceptibility Test

According to the Clinical and Laboratory Standards Institute (CLSI) standards [24], the Kirby-Bauer disc diffusion technique was employed to analyze the antibiotic susceptibility profiles of all *Salmonella* isolates using Oxoid antibiotic discs. Inoculates were made in sterile normal saline and adjusted to the 0.5 McFarland density standard. The test organisms were inoculated on Muller-Hinton agar (Oxoid), subjected to an antibiotic diffusion concentration gradient from an impregnated paper disc, and incubated at 37°C for 24 hours. Ampicillin (10 *μ*g), amoxicillin/clavulanic acid (20/10 *μ*g), ceftriaxone (30 *μ*g), gentamicin (10 *μ*g), meropenem (10 *μ*g), tetracycline (30 *μ*g), ciprofloxacin (5 *μ*g), cotrimoxazole (25 *μ*g), and chloramphenicol (30 *μ*g) were among the antibiotic discs used. According to the CLSI guidelines, the diameters of the zones of inhibition around the discs were measured to the nearest millimeter and classified as sensitive, intermediate, or resistant. *Salmonella* spp., which exhibited resistance to at least one antimicrobial drug in three or more antimicrobial categories, was considered as multidrug-resistant (MDR). *Salmonella* spp. was classified as multidrug-resistant (MDR) if they were resistant to at least one antibiotic in three or more antimicrobial groups [24].

### 2.6. Data Quality Assurance

To maintain the quality of the data, a pretest was conducted at Arba Minch Dilfana Primary Hospital on 5% of the sample size, and appropriate measures have been taken throughout the process of data collection and laboratory work. Standard Operating Procedures (SOPs) were strictly followed at each step. For malaria diagnosis, the working solution of Giemsa was prepared by filtering the crystals. In addition, the glass slides were labeled in such a way that the slide code matched the file of the particular individual. During blood sample collection, one sterile lancet was used per patient, a color Atlas was used during microscopic examination, and the smear was examined by three readers, while in the microbiological aspect, it was verified that the media and reagents met the expiration date and quality control parameters. All culture media were prepared per the manufacturer's instructions, and the sterility of the culture media was tested by incubating 5% of the batch overnight at 35–37°C to assess possible contamination. *Escherichia coli* ATCC 25922 standard control strains obtained from the Ethiopian Public Health Institution were used as quality control for culture media and biochemical tests as per the standard [24].

### 2.7. Ethics Approval and Consent to Participate

Ethical clearance was obtained from the Institutional Review Board of Arba Minch University, College of Medicine and Health Sciences (Ref. No. CMHS/21715/21). Permission was obtained from Arba Minch General Hospital. Study participants were informed about the purpose of the study, their rights to withdraw, and the confidentiality of the obtained information. For participants under the age of 18, informed verbal and written consent was obtained from their parents or guardians prior to the interview. To overcome the issue of COVID-19, all standard precautions were followed, such as wearing a mask and using personal protective equipment when collecting and processing the sample. Significant laboratory results were reported to the respective patients' physicians for treatment.

### 2.8. Statistical Analysis

The Statistical Package for Social Sciences (SPSS) software version 22 was used to analyze the data.Descriptive statistics like frequency, mean, and percentage were calculated. Logistic regression analysis was applied for identifying predictor variables associated with a malaria-typhoid fever coinfection. Bivariate logistic regressions analysis was used to assess associations between malaria-typhoid fever coinfection and potential associated factors. Variables with *P-*value less than 0.25 in the bivariable analysis were jointly entered into a multivariable analysis. The presence of associations and statistically significant was determined at a *P*-value less than or equal to 0.05.

## 3. Results

### 3.1. Sociodemographic Characteristics of the Study Participants

A total of 416 febrile patients participated in the study. About 230 (55.3%) patients were males. The age range of the study participants was from 2 to 68 years old, with a mean (SD) age of 23.4 (11.5) years old. Among the age distribution, 222 (53.4%) of the study participants were in the age group of 12^_^25-year-olds. The majority of the study participants were living in rural areas (54.1%) and unmarried (81.5%). More than half (53.1%) of the study participants were students (Table 1**)**.

### 3.2. Clinical Characteristics of the Study Participants

Among a total of 416 study participants who had fever, 163 (39.2%) had a filling of illness for 8–14 days. Half (215) of the study participants had a history of fever for a week, and around 60% (246) had intermittent fever, while 49.5% (206) of study participants had moderate-grade fever. One hundred thirty-eight (33.2%), 327 (78.6%), and 43 (10.3%) study participants had clinical presentations of chills, fatigue, and convulsion, respectively **(**Table 2**)**.

### 3.3. Magnitude of Malaria, Typhoid, and Coinfection

**Figure 1:**
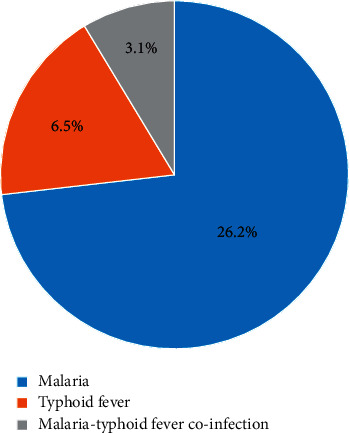
Magnitude of malaria, typhoid fever, and coinfection among febrile patient attending Arba Minch General Hospital, Southern Ethiopia, 2021.

One hundred nine (26.2%) study participants were microscopically confirmed to be malaria positive. From the confirmed malaria cases, 66.0%, 29.4%, and 4.6% of infections were *Plasmodium falciparum, Plasmodium vivax,* and mixed infections (*P. falciparum* and *P. vivax*), respectively, whereas the magnitude of culture-confirmed typhoid fever was 27 (6.5%). Overall, 13 (3.1%) study participants had malaria-typhoid fever coinfection **(**Figure 1**)**.

### 3.4. Predictors to Malaria-Typhoid Fever Coinfection

Clinical manifestations were found to be significantly associated with malaria-typhoid fever coinfection (*P* < 0.05). Patients with continuous patterns of fever were more than five times more likely to have malaria-typhoid coinfection than those who had intermittent patterns of fever (AOR = 5.84; 95% CI: 1.44–23.71, *P*=0.014). Likewise, patients presenting with chills were more than four times more likely to have coinfection with malaria and typhoid (AOR = 3.94; 95% CI: 1.04–14.89, *P*=0.044) (Table 3).

### 3.5. Antibiotic Susceptibility Patterns

We found that the *Salmonella* isolates varied considerably in their susceptibility to all the antimicrobials tested. The high percentage of isolates resistant was observed to ciprofloxacin (51.9%) and ampicillin (55.6%). Likewise, 48% of the isolates showed resistance to tetracycline. On the other hand, 70.4% of *Salmonella* isolates were susceptible to meropenem, and 59.3% of isolates were susceptible to amoxicillin/clavulanic acid, gentamicin, and chloramphenicol. The overall MDR result obtained from the present study was 29.6% (*n* = 8) (Table 4).

## 4. Discussion

In this study, the rate of microscopically confirmed malaria infection in the study area was 26.2% (95% CI: 21.7, 30.5). The overall result is comparable to the study conducted in Ethiopia (22.1%), Nigeria (22.2% to 27%), and Burkina Faso (30.4%) [9–11,25]. However, studies conducted at Arba Minch, Ziway Health Center, and the Amibara District Hospital in Ethiopia and reports from Nigeria and India showed a lower prevalence of malaria ranging from 2.5 to 21.0% [4,12,13,18,26,27]. The higher malaria prevalence in this study could be attributed to differences in the sample blood used and the study participants. In the present study, capillary blood smear was used, whereas the previous studies conducted in Ethiopia and other African countries used EDTA venous blood [12,13]. On the other hand, the study employed in Amibara District Hospital [4] was a community-based study design, while this study used an institution-based design.

On the other hand, the rates of malaria detection from Northwest Ethiopia (36.5%) [14], Cameron (56.8%), Nigeria (54.2% and 80.8%), and Pakistan [14,15,28–30] were comparatively much higher than our results. The lower prevalence of malaria parasitemia in our study might be due to seasonal variation since the transmission rate of malaria in Ethiopia is unstable and varies from season to season as it varies with temperature and rainfall, which are important limiting factors for transmission. In addition, it might be due to a diagnostic method difference [30].

The prevalence of culture-confirmed typhoid fever was 6.5% [95% CI: (4.3–9.1)]. This finding is more or less similar to previous studies done in Shashemene, Ethiopia (5.0%) [22], West, Ghana (6.37%) [31], Northern, Uganda (6.8%) [20], and Kolkata, India (4.5%) [27]. The prevalence is much lower than the extent of prevalence stated in Northern Ethiopia (68.7%) (17), Nigeria (12%–40%) [32], Northern India (22%) [33], and Karnataka, India (55.4%) [34]. Nevertheless, the rate of typhoid fever infection currently observed was relatively higher than previous reports from Ethiopia (1.6% and 2.6%) [17,19], and from other African countries (0% to 2.6%) [28,35,36], and study reports from India (1.99% and 4%) [18,37]. The higher typhoid fever infection rate observed in our study could be attributed to variations in the amount of sample blood used, the diagnostic methods, sociodemographic characteristics, the degree of sanitation and hygiene among study populations, and variations in the study period. For instance, studies done in Ethiopia [19] and Northwest Nigeria [35] used a small volume of blood samples where the most important factor that determines the success of the blood culture is the volume of the blood processed. There is a 40% rate of cultures positive for the organism if 20 ml rather than 10 ml of blood is cultured [35]. In addition, an increase in blood volume increases sensitivity for typhoid diagnosis from 51% for 2 mL of blood to 65% for 10 mL, or by 3% for each additional milliliter [26].

Out of 416 individuals tested, 3.1% [95% CI: (1.7–4.9)] were positive for malaria-typhoid fever coinfection. Of these, 7 (53.8%) were coinfected with *P. falciparum*, 4 (30.8%) were with P. vivax, and 2 (15.4%) were with mixed infections (*P. falciparum* and *P. vivax*). This finding agrees with the results reported for Ghana (1.91%) [38], India (1.6% and 1.59%) [33,36], and Pakistan (4%) [30]. In contrast, the current coinfection prevalence is lower than reported in KMC&H, Guntur (6.5%) [39], Cameroon (7.8%) [15], Enugu, Nigeria (15.6%) [31], Calabar, Nigeria (28%) [21], Southeastern Nigeria (36.2%) [28], and Akure, Nigeria (51.5%) [40]. The low prevalence of malaria-typhoid fever coinfection in our study might be due to the test methods employed for the diagnosis of malaria and typhoid fever. For instance, studies conducted in India [33,37] involved multiple diagnostic methods to screen and confirm typhoid fever infection, involving blood culture, stool culture, and urine culture, and they were confirmed by PCR technique, while in our study, only blood culture was used. Similarly, the study done in Pakistan [30] used a PCR technique for malaria identification. On the other hand, the prevalence of coinfection identified in our study is higher than that reported in Northwest, Ethiopia (0.5%) [14], North-West Nigeria (0%) [36], Kaduna state, Nigeria (0.5%) [9], Northern India (1.1%) [33], New Delhi (1.59%) [37], and Uttar Pradesh, India (1%) [18].

Clinical features, such as continuous patterns of fever and chills, were found to be statistically significant with malaria-typhoid fever coinfection (*P* < 0.05), which is in contrast to a study done in Northwest Ethiopia [14]. These significant associations of patterns of fever and chills with coinfection may be able to aid in the differential diagnosis during physical examination of coinfection. However, there were no sociodemographic or other clinical and behavioral factors significantly associated with the coinfection, which agrees with a study in Northwest Ethiopia [14]. In fact, the presence of coinfection in this study was high among males and rural residents. The reason for male and rural resident participants' preponderance could be attributed to a higher degree of exposure as a result of their occupational, domestic, and leisure activities as compared to female as well as urban residents. It is also possible that the participant's age influences the prevalence and severity of coinfection. We observed that schoolchildren had a greater rate of coinfection; these findings are consistent with earlier research [4–7,15,30].

In the present study, a high degree of resistance among isolated *Salmonella* spp. was found, particularly against ampicillin, ciprofloxacin, and tetracycline antibiotics. This finding is in line with the studies performed on the drug resistance patterns of *Salmonella* spp. from different regions of Ethiopia [17,41]. According to the WHO, MDR exhibited by species of *Salmonella* is currently a serious global concern. In the present study, 29.6% of the isolates were found to be MDR, which is lower than that reported in the previous study done in Ethiopia (50% [42]. This level of MDR is likely to be correlated with high self-medication practice, inappropriate antibiotic prescriptions, and the long-term use of those antibiotics. On the other hand, a notable result of the present study is that more than 59.3% of isolates were susceptible to meropenem, amoxicillin/clavulanic acid, gentamicin, and chloramphenicol. The possible justification for the susceptibility shown by *Salmonella* isolates to these antibiotics could be due to lack of exposure to these drugs arising from the relatively high cost and unavailability of meropenem and prescribers' preference, indicating the possibility of using these drugs to manage enteric fever in the study setting of Arba Minch General Hospital.

## 5. Conclusions

This study showed that the prevalence of microscopically confirmed malaria, culture-confirmed typhoid fever, and its coinfection in febrile patients was comparable with the previous findings. Continuous patterns of fever and chills were statistically associated with malaria-typhoid fever coinfection, which may aid during differential diagnosis of malaria-typhoid coinfection. The overall prevalence of malaria was higher than typhoid fever. More than twenty-nine percent of *Salmonella* isolates were found to be MDR. The overall results indicate that malaria–typhoid fever coinfection is a health problem in the study area. With the consideration of higher prevalence of drug resistance of *Salmonella* spp. and higher prevalence of malaria–typhoid fever coinfection, proper diagnostic procedure should be implemented for proper use of drugs.

### 5.1. Limitation of the Study

The limitations of the present study include the cross-sectional study design with a relatively small sample size and a short study period in hospital-based study that enrolled participants in the outpatient department. In addition, hematological profiles (white blood cells, red blood cells, and monocytes ratio) of febrile patients as well as the prognosis of the patient were not considered in this study due to budget and resource constraints.

## Figures and Tables

**Table 1:** Socio-demographic characteristics by coinfection of febrile patient (*n* = 416) at Arba Minch General Hospital, Southern Ethiopia, 2021.

Variables	Category	Coinfection	
		No (%)	Yes (%)
Sex	Male	219 (95.2)	11(4.8)
	Female	184 (98.9)	2 (1.1)

Age (year)	2–5	44 (91.7)	4 (8.3)
	6–11	51 (94.4)	3 (5.6)
	12–25	218 (98.2)	4 (1.8)
	26–45	79 (98.8)	1 (1.2)
	≥46	11 (91.7)	1 (8.3)

Residence	Urban	189 (99.0)	2(1.0)
	Rural	214 (95.1)	11(4.9)

Marital status	Married	73 (94.8)	4 (5.2)
	Unmarried	330 (97.3)	9 (2.7)

Education status	Illiterate	7 (87.5)	1 (12.5)
	Kindergarten (KG)	26 (89.7)	3 (10.3)
	1 school	126 (96.9)	4 (3.1)
	2 school	166 (97.6)	4 (2.4)
	College & above	78 (98.7)	1 (1.3)

Occupation	Housewife	5 (83.3)	1(16.7)
	Farmer	34 (70)	2 (30)
	Student	215 (97.3)	6 (2.7)
	Merchant	78 (97.5)	2 (2.5)
	Employee	55 (98.2)	1(1.8)
	Labour worker	16 (94.1)	1 (5.9)

**Table 2:** Clinical characteristics by Coinfection of febrile patient (*n* = 416) at Arba Minch General hospital, southern Ethiopia, 2021.

Variables	Category	Coinfection	
		No (%)	Yes (%)
Duration of illness	1–7 days	138 (92.6)	11 (7.4)
	8–14 days	162 (99.4)	1 (0.6)
	15–21 days	103 (99.0)	1 (1.0)

Duration of fever	≤ a week	206 (95.8)	9 (4.2)
	Within two weeks	182 (98.4)	3 (1.6)
	More than two weeks	15 (93.8)	1 (6.2)

Pattern fever	Continuous	161 (94.7)	9 (5.3)
	Intermittent	242 (98.4)	4 (1.6)

Degree of fever	Mild (100.5–102.1°F)	56 (98.2)	1 (0.8)
	Moderate (102.2–104.0°F)	203 (98.5)	3 (1.5)
	High (104.1–106.0°F)	105 (95.5)	5 (4.5)
	Hyperpyrexia (>106.0°F)	39 (90.7)	4 (9.3)

Fatigue	Yes	317 (96.9)	10 (3.1)
	No	86 (96.6)	3 (3.4)

Headache	Yes	277 (96.9)	9 (3.1)
	No	126 (96.9)	4 (3.1)

Chills	Yes	130 (94.2)	8 (5.8)
	No	273 (98.2)	5 (1.8)

Vomiting	Yes	167 (96.0)	7 (4.0)
	No	236 (97.5)	6 (2.5)

Diarrhea	Yes	59 (96.7)	2 (3.3)
	No	344 (96.9)	11(3.1)

Convulsion	Yes	39 (90.7)	4 (9.3)
	No	364 (97.6)	9 (2.4)

**Table 3:** Magnitude of malaria-typhoid fever Coinfection in relation to sociodemographic characteristics and clinical features in febrile patient at Arba Minch General Hospital, Southern Ethiopia, 2021.

Variables	Categories	Coinfection (%)	Corollary (95% CI)	AOR (95% CI)	*P* value
Gender	Male	11 (4.8)	4.62 (1.01–21.11)	3.94 (0.80–19.44)	0.092
	Female	2 (1.1)	1	1	

Residence	Urban	2 (1.0)	1	1	
	Rural	11 (4.9)	4.86 (1.06–22.19)	4.32 (0.86–21.64)	0.075

Duration of illness	1–7 days	11 (7.4)	8.21 (1.04–64.61)	5.29 (0.62–44.87)	0.127
	8–14 days	1 (0.6)	0.64 (0.04–10.28)	0.39 (0.02–7.13)	0.522
	15–21 days	1 (1.0)	1	1	

Pattern fever	Continuous	9 (5.3)	3.38 (1.02–11.17)	5.84 (1.44–23.71)	0.014^*∗*^
	Intermittent	4 (1.6)	1	1	

Degree of fever	Mild (100.5–102.1°F)	1 (0.8)	1	1	
	Moderate (102.2–104.0°F)	3 (1.5)	0.83 (0.084–8.11)	0.97 (0.09–10.0)	0.981
	High (104.1–106.0°F)	5 (4.5)	2.67 (0.304–23.39)	2.76 (0.28–26.5)	0.379
	Hyperpyrexia (>106.0°F)	4 (9.3)	5.74 (0.618–53.36)	4.93 (0.49–49.1)	0.174

Chills	Yes	8 (5.8)	3.36 (1.08–10.47)	3.94 (1.04–14.89)	0.044^*∗*^
	No	5 (1.8)	1	1	

Convulsion	Yes	4 (9.3)	4.15 (1.22–14.10)	4.13 (0.99–17.28)	0.052
	No	9 (2.4)	1		

Note. ^*∗*^Statistically significant. AOR: adjusted odds ratio; CI: confidence interval; COR: crude odds ratio.

**Table 4:** Antibiotic Susceptibility Pattern of *Salmonella* isolates (*n* = 27) among Febrile Patient Attending Arba Minch General Hospital, Southern Ethiopia, 2021.

Antimicrobial agent	Susceptible	Intermediate	Resistant
	No (%)	No (%)	No (%)
Ampicillin	9 (33.3)	3 (11.1)	15 (55.6)
Amoxicillin/clavulanic acid	16 (59.3)	3 (11.1)	8 (29.6)
Ceftriaxone	12 (44.4)	7 (25.9)	8 (29.6)
Gentamicin	16 (59.3)	4 (14.8)	7 (25.9)
Meropenem	19 (70.4)	3 (11.1)	5 (18.5)
Tetracycline	10 (37)	4 (14.8)	13 (48)
Ciprofloxacin	9 (33.3)	4 (14.8)	14 (51.9)
Cotrimoxazole	15 (55.6)	3 (11.1)	9 (33.3)
Chloramphenicol	16 (59.3)	3 (11.1)	8 (29.6)

## Data Availability

The data sets studied in this work are only available from the principal author upon reasonable request.
